# Physical activity and glycosylphosphatidylinositol-specific phospholipase D1 (GPLD1) plasma levels in different age cohorts

**DOI:** 10.1371/journal.pone.0349883

**Published:** 2026-06-01

**Authors:** Ghulam Shere Raza, Jari Jokelainen, Ville Stenbäck, Nalini Sodum, Toni Karhu, Dominique D. Gagnon, Juhani Leppäluoto, Marjo-Riitta Järvelin, Sirkka Keinänen-Kiukaanniemi, Karl-Heinz Herzig

**Affiliations:** 1 Research Unit of Biomedicine and Internal Medicine, Medical Research Center, Oulu University Hospital, Biocentre of Oulu, University of Oulu, Oulu, Finland; 2 Research unit of Population Health, Faculty of Medicine, University of Oulu, Oulu, Finland; 3 Unit of Primary Care, Oulu University Hospital, Oulu, Finland; 4 Faculty of Sports and Health Sciences, University of Jyväskylä, Jyväskylä,‌‌ Finland; 5 Clinic for Sports and Exercise Medicine, Department of Sports and Exercise Medicine, Faculty of Medicine, University of Helsinki, Helsinki, Finland; 6 MRC Centre for Environment and Health, Department of Epidemiology and Biostatistics, ‌‌School of Public Health, Imperial College London, London, United Kingdom; 7 Unit of General Practice, Oulu University Hospital, Oulu, Finland; 8 Pediatric Gastroenterology and Metabolic Diseases, Pediatric Institute, Poznan University of Medical Sciences, Poznań, Poland; Trinium Woman's Hospital, KOREA, REPUBLIC OF

## Abstract

Physical activity (PA) has numerous positive health effects, including improvements in cognitive function. PA stimulates the release of glycosylphosphatidylinositol-specific phospholipase D1 (GPLD1) from the liver, resulting in hippocampal neurogenesis in mice and improved cognition. Interestingly, PA also increases plasma GPLD1 in healthy humans, suggesting a role in cognition, but regulatory factors modulating its concentrations remain unclear. We investigated whether different PA modes, diseases (obesity, hypertension, and diabetes), and age affect plasma GPLD1 levels using three different study cohorts. Plasma GPLD1 levels were analyzed in three studies cohorts comprised of 1) young adults (25 yrs) performing moderate exercise, 2) elderly (68.9 yrs) and 3) of prediabetic subjects (59.4 yrs) performing different habitual PA. We found that prediabetic subjects had higher plasma GPLD1 levels than elderly and young adults. Elderly with diabetes showed significantly higher GPLD1 levels than non-diabetic subjects. In elderly, plasma GPLD1 correlated negatively with average steps, but there was no association after taking their diabetes status into account. GPLD1 exhibited a positive correlation with age in elderly with normal glucose tolerance, and with BMI in subjects with impaired glucose tolerance. Average step numbers negatively correlated with BMI and diastolic BP in the elderly, and BMI in prediabetic subjects. BMI was positively correlated with both diastolic and systolic BP in elderly subjects, and with diastolic BP in young adults. In conclusion age and diabetes affect plasma GPLD1 levels in humans. Age is one of the determining factors, as moderate exercise in young adults did not change plasma GPLD1 levels. Habitual physical activity did not alter plasma GPLD1 levels in individuals with prediabetes or diabetes. The release of GPLD1 into the blood and its mechanistic influences on cognitive functions would need further investigation in humans.

## Introduction

**Fig 1:**
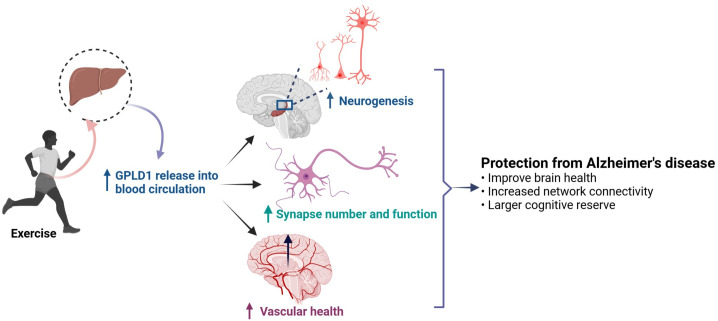
Hypothesis: Exercise increases GPLD1 secretion from the liver, which improves vascular health, hippocampal neurogenesis, and cognition.

Physical exercise improves metabolic, cardiovascular, immune, and neurological health [1]. Physical activity (PA) specifically contributes to preserve cognition across the lifespan and reduces the risk of dementia [2,3]. In our globally aging societies, dementia is one of the major disability causes with substantial challenges. Worldwide, about fifty-seven million people had dementia in 2021 with 10 million new cases every year (WHO 2025). Several meta-analyses showed that higher levels of PA decreased risk of cognitive decline [4–6]. In addition, exercise improves memory tasks and cognition in the elderly already with cognitive impairment [7], glucose intolerance [8], and Alzheimer’s disease [9]. Northey et al. demonstrated that regular exercise improves cognitive function in subjects over 50 years regardless of cognitive status [10]. Exercise induces secretion of several factors such as myokines from skeletal muscles, adipokines from adipose tissue, hepatokines from the liver, and neurokines from neurons [1]. Recently, Horowitz et al. found that increased PA stimulates the release of glycosylphosphatidylinositol-specific phospholipase D1 (GPLD1) from the liver to the blood circulation, resulting in hippocampal neurogenesis and improved cognitive functions in aged mice and humans [11] (Fig 1).

GPLD1 does not cross the blood-brain barrier but changes the urokinase-type plasminogen activator receptor (uPAR) signaling pathway with its downstream targets. GPLD1 is a 110–120 kDa protein abundant in mammalian plasma associated with high-density lipoproteins (HDL) [12]. It is an 815-amino acid enzyme, mainly produced in the liver and pancreatic islets, and minor amounts in the brain, muscle, kidney, immunocytes, and inflammatory cells [13,14]. GPLD1 cleaves glycosylphosphatidylinositol (GPI) anchored proteins, releasing the attached protein from the plasma membrane [15]. GPI-anchored proteins are integral membrane proteins on the cell surface, lacking a transmembrane domain, and act as transcytotic transporters [16]. GPLD1 levels increased in circulation after the onset of diabetes and insulin resistance in rats and humans, suggesting its association with diabetes [17,18]. In addition, GPLD1 upregulates cytokine expression, which might play a role in inflammation [13]. Other modifiable factors for GPLD1 may include diabetes and obesity [9,19], which have not yet been investigated.

We hypothesize that GPLD1 levels might be affected by age, diabetes, and obesity in addition to different modes of PA. We therefore investigated the release of GPLD1 using three different cohorts of different ages, degrees of obesity, diabetes, and levels of physical activity. Our aim was to study the effect of exercise/daily habitual activity on plasma GPLD1 in different ages. Our aim is not to compare these cohorts, but to investigate the effect of exercise/PA in different age and disease status on plasma GPLD1 levels.

## Materials and methods

The study population consisted of individuals from three study population with similar ethnic and cultural backgrounds and even comparable lifestyles from the same region in Northern Finland as discussed below. Study population 1 consisted of young adults (25 yrs), study population 2 consisted of elderly (68.9 yrs) Oulu-1945 birth cohort and study population 3 of prediabetic subjects (59.4 yrs), who performed exercise or habitual PA. Blood samples from young adults and prediabetic subjects were collected in the morning between 8 and 10 am after a 12-hour fast at the beginning and end of the intervention. In elderly overnight (12 hrs) fasted blood samples were collected for measurement of plasma parameters. Plasma GPLD1 was measured in young adults (n = 19) performing medium session exercise at thermoneutral (TN) temperature, prediabetic subjects (n = 38) with daily steps <3000 and > 6000 steps and elderly subjects (n = 526). The plasma samples from the study populations were archived at university of Oulu and GPLD1 measurement was started on 15.04.2024. The authors had access to the information that could identify individual participants after data collections. Plasma levels of GPLD1 were measured by Enzyme-Linked Immunosorbent Assay (ELISA) using the commercial human GPLD1 ELISA kit (HUFI01252: AssayGenie, Dublin 1, Ireland, UK) according to the manufacturer’s instructions. All the samples were measured in duplicates with internal controls and blinded data.

### Ethics declarations

All the participants provided written informed consent. The studies were approved by the Regional Medical Research Ethics Committee of the Wellbeing Services County of North Ostrobothnia, Oulu Finland. All the studies were performed in accordance with the Declaration of Helsinki.

### Study population 1: Young adults

Healthy young adults (24.6 ± 3.3 yrs, n = 37) participated and were randomly assigned into the cold (n = 18) and thermoneutral (n = 19) groups. The recruitment period for young adults was August 2020 – September 2020. Three subjects from the cold group dropped out during the training period due to personal issues. The participants did not perform additional exercises during the study period. Blood samples were collected from all the participants before and 48–96 hrs after the last exercise bout (short, medium, and long) session. The participants were asked to refrain from alcohol, nicotine products, caffeine, and vigorous exercise 24 hours before their measurements. The dietary and fluid intake for 24 hours before pre-training and post-training measurements were identical. Pre- and post-training measurements were performed on the same day ± 2 hours. Anthropometric parameters were recorded, and body fat percent was measured by bioimpedance analysis scale (Omron HBF-514C, Omron Healthcare Co., Ltd., Kyoto, Japan) as shown in S1 Table in S1 File. The participants completed one short, one medium, and one long training session per week in a self-determined training order for 7 weeks as shown in Table 1.

**Table 1:** Exercise protocol for study population 1: young adults.

	Session	Intensity (% Wmax)	Duration	Rest	Repetition
Weeks 1–2	Long Medium Short	80% 90% 100%	5 min 2 min 30 sec	2.5 min 2 min 30 sec	3 4 6
Weeks 3–4	Long Medium Short	80% 90% 105%	6 min 2 min 30 sec	3 min 2 min 30 sec	3 6 8
Weeks 5–6	Long Medium Short	85% 95% 120%	6 min 2 min 40 sec	3 min 2 min 20 sec	4 8 10
Weeks 7	Long Medium Short	85% 95% 130%	7 min 2 min 40 sec	3.5 min 2 min 20 sec	4 10 12

W_max=_ maximal cycling power in the VO2 peak test.

Each training session was performed on a different day with one rest day between each training session. The exercise intensity and duration were increased every 2 weeks and subjects performed 3 high-intensity training sessions per week for 21 training sessions. The cold group completed their training sessions in a climate chamber (Arctest Oy, Espoo, Finland) set at 0 °C and 50% relative humidity and the thermoneutral group trained at room temperature (approximately 22 °C). Training sessions were done on a cycle ergometer. If participants could not finish the training sessions with the prescribed intensity, the intensity was decreased so that participants could complete the session.

### Study population 2: Elderly (Oulu-1945) birth cohort

**Fig 2:**
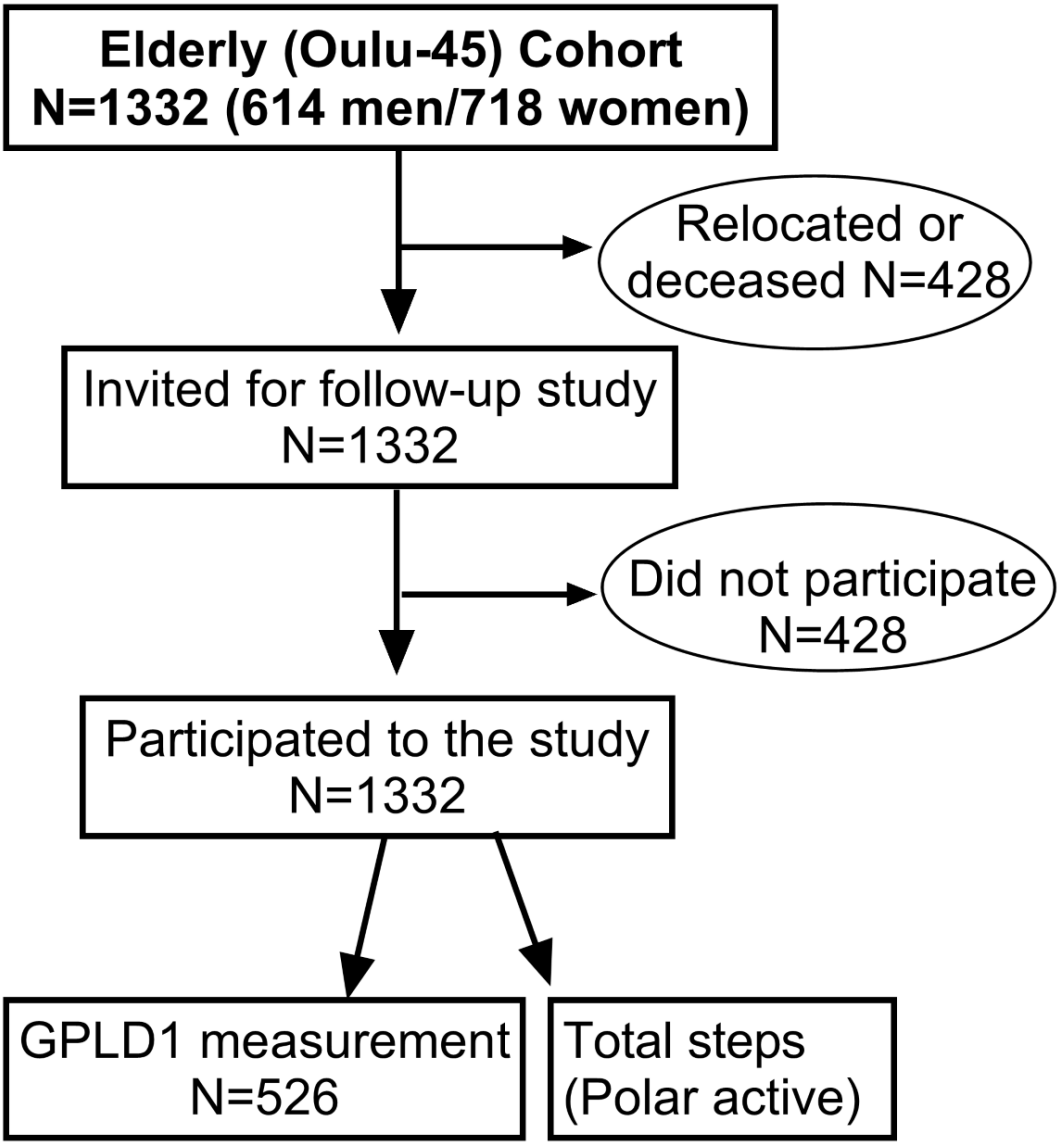
Flow chart of the study design for study population 2: elderly (Oulu-1945) birth cohort.

The study population consisted of individuals born in 1945 in the City of Oulu, Finland (Oulu-1945 birth cohort) [20]. 904 individuals from the original 1945 cohort were invited for a follow-up study from April 2013–June 2015 (Fig 2).

Exclusion criteria were BMI over 40 kg/m^2^ or irregular heart rate. Anthropometric data and clinical data of the subjects are shown in S2 Table in S1 File. The participants took part in two study visits. During the first visit, overnight fasted blood samples were drawn and bioelectrical impedance analysis (BIA) (InBody 720, InBody, Seoul, Korea) was conducted. In the second visit, an oral glucose tolerance test (OGTT) was performed. Participants were diagnosed as type-2 diabetic (T2D) if either they were taking diabetes medication, or had a fasting blood glucose (FBG) value ≥7.0 mmol/L, or the 2h BG value ≥11.1 mmol/L. Participants were categorized as impaired glucose metabolism if either they had increased fasting blood glucose (6.1–6.9 mmol/L) (IFG) or an increased 2h blood glucose (7.8–11.1 mmol/L) (IGT) in OGTT. All the other participants were regarded as having normal glucose tolerance (NGT). The participants filled in questionnaires regarding their lifestyle, mental status, and perceived health. Smoking history and present status were inquired by questionnaires and results were categorized into three groups: Non-smokers, former smokers (> 6 months), and smokers. Alcohol consumption was evaluated by frequency, type and amount and translated into grams of alcohol/day. At the end of the second visit, the participants were given an activity meter (Polar Active, Polar Electro, Finland) to measure physical activity. The habitual physical activity of the participants was measured with a wrist-worn acceleration meter (Polar Active, Polar Electro, Finland) [21]. The participants wore the activity meter during the wakeful time for two weeks and activity was recorded.

### Study population 3: Prediabetes subjects

The study population and experimental design have been described previously [22,23]. Briefly, the study population consisted of 68 late middle-aged sedentary and prediabetic subjects who participated in a 3-month randomized controlled trial (RCT) in the city of Oulu, Finland. Participants were recruited for the study between November 2009 and January 2010 from the outpatient diabetic clinics in Oulu. All subjects were evaluated for T2D with the FINDRISC questionnaire and those receiving scores >15 underwent an oral glucose tolerance test. The participants selected for the study met the WHO criteria for impaired fasting glucose (≥ 5.6 and < 7.0 and 2 h glucose < 7.8 mmol l^-1^) or impaired glucose tolerance (fasting glucose < 7.8 and 2 h glucose ≥ 7.8 and < 11.1 mmol l^-1^). They were assigned to intervention (n = 33) and control (n = 35) groups. Height, weight, and waist circumference were measured using a standardized protocol and body mass index was calculated. The ages and body weights of the subjects were 58.1 ± 9.9 years and 92.4 ± 19.4 kg in the intervention group and 59.5 ± 10.8 years and 84.6 ± 14.4 kg in the control group as shown in S3 Table in S1 File. Both groups received detailed information on the effect of PA on metabolism in a combined lecture.

The intervention group participated then in supervised exercise sessions three times a week while the control group did not. Each session started with 5 min of stretching as a warm-up and a 20 min walk at an average speed of 3–4 km h^-1^. This was followed by a 5 min stretching and balance exercise, 20 min of walking and 10 min stretching and balance training at the end of the session. After 1.5 months, stretching and balance training between the walking exercises was removed and the walking time was increased to 45 min. The intervention group aimed to meet the ACSM 2011 guidelines of at least 150 min of moderately vigorous PA weekly. However, the subjects were overweight/obese and most of them could not walk at a speed of 5 km h^-1^ required for moderate PA. The habitual PA was measured using an accelerometer (Newtest Exercise Monitor, Newtest, Oulu, Finland) on a belt close to the right iliac crest as described earlier [22,23]. The device was used during waking hours, except during aquatic activities, every day for three months to record the number of steps. Weighted averages of accelerations in walking or running speeds of 3, 6, and 9 km h^-1^ were responsible for 92% of the variance in the energy expenditure. The whole-day wear time of the accelerometer varied between 24–93 days, and the median value was 78 days. The mean number of daily steps in the intervention group was 5870 ± 3277, and in the control, group was 4034 ± 3460 steps at low acceleration levels.

### Statistical analyses

Continuous variables of the study populations are summarized as means with standard deviations (SD), while categorical variables are presented as counts and percentages. Pearson’s chi-squared test was employed to assess differences in proportions between categorical variables. For comparisons of clinical characteristics, non-parametric tests such as the Mann–Whitney U test or Kruskal–Wallis test were used as appropriate. OGTT profiles of elderly (Oulu-1945) subjects include the mean (SD) and median [Q1, Q3] for GPLD1. The statistical test ANOVA was used for the analysis of difference in mean, and Kruskal-Wallis test for the median.

Bivariate correlations (Spearman’s rho) were calculated to explore the relationship between GPLD1 levels and physical activity (measured by daily steps) in the elderly and prediabetic subjects. Additionally, the effect of exercise training on plasma GPLD1 concentrations was examined in young adults. A p-value < 0.05 was considered statistically significant. All analyses were conducted using SPSS software, version 27 (SPSS, Inc., Chicago, Illinois).

## Results

Body mass index (BMI), sex, and age significantly differed between the young adults (25 yrs), elderly (Oulu-45) (68.9 yrs), and prediabetic subjects (59.4 yrs) as analyzed by Pearson’s chi-squared test (Table 2).

**Table 2:** Continuous variables are summarized as means with standard deviations (SD), while categorical variables are presented as counts and percentages.

	Young adults mean age–25 yrs (n=19)	Elderly (oulu-45) mean age – 70 yrs (n=526)	Prediabetes mean age – 59 yrs (n=38)	p values
Sex:				0.027
Male	9 (47.4%)	225 (42.8%)	8 (21.1%)	
Female	10 (52.6%)	301 (57.2%)	30 (78.9%)	
Age	24.4 (3.45)	68.9 (0.54) **	59.4 (10.5) **	<0.001
BMI	26.2 (3.35)	27.7 (4.71)	32.3 (5.33) **	<0.001
GPLD1 μg/ml	2.06 (0.21)	2.43 (0.44)	3.24 (0.55) **	<0.001

Participants of the Oulu-45 cohort were older than prediabetic subjects, but their BMI (27.7 kg/m^2^) was significantly lower than that of the prediabetic subjects (32.3 kg/m^2^). Elderly subjects with T2DM either screen detected (ScT2DM) or prevalent (PrevT2DM) had higher BMI compared to NGT (Table 3).

**Table 3:** Descriptive statistics of the Oulu‑45 cohort participants (n = 526) stratified by OGTT status.

	NGT (n = 275)	IFG/IGT (n = 172)	scT2DM/PrevT2DM (n=79)	p values
Sex:				0.019
Men	108 (39.3%)	72 (41.9%)	45 (57.0%)	
Women	167 (60.7%)	100 (58.1%)	34 (43.0%)	
Age	69.0 (0.56)	68.9 (0.51)	68.9 (0.50)	0.441
BMI	26.1 (3.63)	28.4 (4.74)	30.9 (5.25)	<0.001
GPLD1 μg/ml	2.38 (0.40)	2.44 (0.41)	2.60 (0.50)	<0.001
GPLD1 /μg/ml	2.32 [2.06;2.62]	2.35 [2.13;2.72]	2.51 [2.26;2.83]	0.001

NGT- normal glucose tolerance, IFG-Impaired fasting glucose, IGT- Impaired glucose tolerance, ScT2DM- screen detected type-2 diabetes, PrevDM- known type-2 diabetes

**Fig 3:**
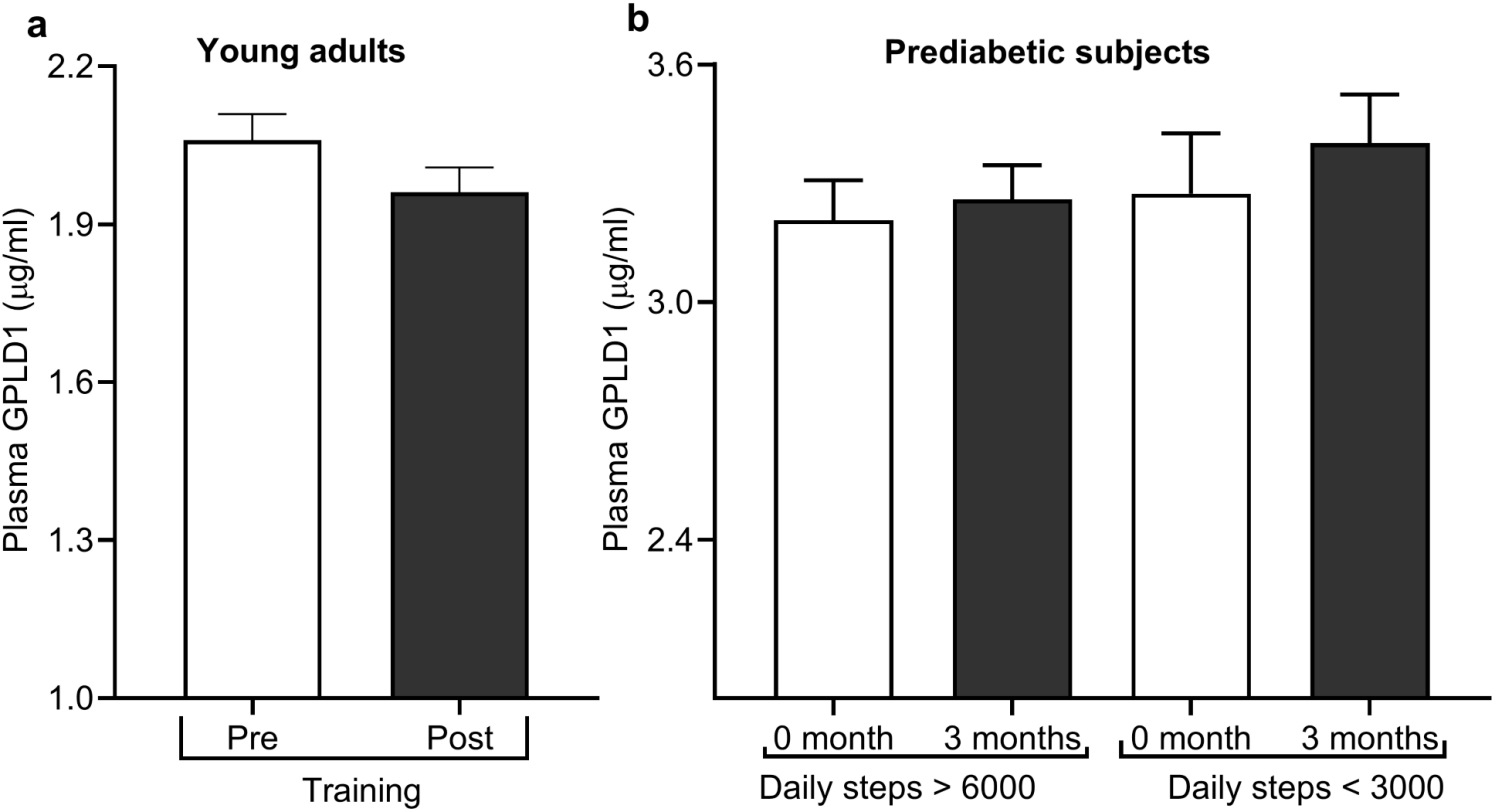
Plasma GPLD1 levels in healthy young adults and prediabetic subjects. Prediabetic subjects had significantly higher GPLD1 compared to healthy young adults. No significant differences were observed in GPLD1 levels before and after training in young adults. Prediabetic subjects with < 3000 steps daily showed higher plasma GPLD1 compared to those with daily steps > 6000, but this was not statistically significant.

The plasma GPLD1 concentrations in the three cohort were 2.25–4.42 μg/ml with an inter- and intra-assay CV of 4% and 7.6% respectively. Plasma GPLD1 levels were significantly higher in prediabetic subjects compared to young adults and elderly (Oulu-45) as analyzed by the Mann–Whitney U test or Kruskal–Wallis (Table 2). Elderly with IGT showed higher plasma GPLD1 as shown mean (SD) and median [Q1, Q3] compared to NGT but there was no difference in plasma GPLD1 between NGT and impaired fasting glucose (IFG) (Table 3). Elderly with T2DM either scT2DM or PrevDM subjects showed significantly higher GPLD1 compared to NGT (Table 3). Post-exercise GPLD1 levels were lower in young adults than pre-exercise (Fig 3).

Physical activity intervention for 3 months increased plasma GPLD1 in prediabetic subjects, however, subjects performing < 3000 steps daily showed higher plasma GPLD1 compared to daily steps > 6000, but these were not statistically significant (Fig 3). Spearman and Pearson correlation analyses in young adults, elderly, and prediabetic subjects are shown (Table 4).

**Table 4:** Spearman correlation analysis in young adults, elderly (Oulu-45 cohort), and prediabetic subjects.

	GPLD1	Age	BMI	Syst BP	Diast BP
**Young adults (n = 19)**					
GPLD1	1.000				
Age	0.277	1.000			
BMI	0.443	0.194	1.000		
Syst BP	0.298	−0.004	0.347	1.000	
Diast BP	0.191	0.197	0.461*	0.840***	1.000
**Elderly (Oulu-45 Cohort, n = 526)**					
GPLD1	1.000				
Age	0.030	1.000			
BMI	0.129**	−0.079	1.000		
Syst BP	−0.007	−0.028	0.109*	1.000	
Diast BP	−0.027	−0.035	0.188***	0.688***	1.000
Avg_Steps	−0.144**	−0.066	−0.273***	−0.022	−0.099*
**Prediabetic subjects (n = 38)**					
GPLD1	1.000				
Age	0.099	1.000			
BMI	0.145	−0.539***	1.000		
Syst BP	0.071	−0.046	−0.120	1.000	
Diast BP	0.070	−0.128	−0.222	0.694***	1.000
Avg_Steps	−0.034	0.318	−0.433**	−0.024	0.085

In elderly subjects (Oulu-45), GPLD1 levels were positively correlated with BMI and negatively correlated with average step numbers as calculated by Spearman’s rho and Pearson correlation analysis (Table 4). However, in young adults and prediabetic subjects, no significant correlations were observed between GPLD1 and BMI and GPLD1 and daily steps. BMI was positively correlated with diastolic and systolic blood pressure (BP) in elderly subjects and diastolic BP in young adults. In addition, the average step numbers negatively correlated with BMI and diastolic BP in elderly subjects and BMI in prediabetic subjects. In elderly average steps negatively correlated with BMI which was independent of their diabetes status (Table 5).

**Table 5:** Spearman correction in elderly based on glucose tolerance status.

	GPLD1	Age	BMI	Syst BP	Diast BP
**Elderly (Oulu-45 cohort) subset_(NGT 275)**					
Age	0.143*				
BMI	−0.057	−0.003			
Syst BP	−0.051	−0.093	0.075		
Diast BP	−0.024	−0.068	0.172**	0.727***	
Avg_Steps	−0.049	−0.075	−0.253***	0.025	−0.087
**Elderly (Oulu-45 cohort) subset_IFG-IGT (172)**					
Age	0.07				
BMI	0.190*	−0.12			
Syst BP	−0.136	0.017	0.013		
Diast BP	−0.026	−0.008	0.286***	0.631***	
Avg_Steps	−0.142	−0.063	−0.196*	−0.016	−0.088
**Elderly (Oulu-45 cohort) subset_scDM-PrevDM (79)**					
Age	−0.128				
BMI	0.112	−0.079			
Syst BP	0.124	−0.051	0.029		
Diast BP	−0.174	−0.023	0.086	0.566***	
Avg_Steps	−0.185	−0.049	−0.253*	−0.008	−0.092

In young adults, habitual steps were not measured; their training consisted of significant aerobic exercise/training using an ergometer. GPLD1 did not correlate with average step in elderly when the data were normalized to their glucose tolerance status shown in Table 5. GPLD1 positively correlated with BMI in elderly with IFG/IGT but not in NGT and T2DM subject (Table 5). In addition, plasma GPLD1 showed positive correlations with the age in elderly with NGT indicating that plasma GPLD1 is increased with age.

## Discussion

Horowitz et al. reported an increase in plasma GPLD1 levels with average daily steps > 7100 in healthy subjects (age 73.7 yrs; BMI 26.6; step range 930–16270) [11]. We found that plasma GPLD1 negatively correlates with avg. daily steps in elderly, but not in young adults and prediabetes subjects. However, the negative correlation lost its significance after analyzing the elderly subject with their diabetes status (Table 5), indicating that diabetes affects plasma GPLD1 levels. The different findings in our study could be due to the number of daily average steps and their intensity, measured with a different validated accelerometer [24,25]. In our study, exercise and physical activity did not change plasma GPLD1 levels in young adults under moderate-intensity training for 7 weeks and prediabetic subjects with average daily step numbers of > 6000 for 3 months. Recently, Ren et al. demonstrated that wheel running 3 times/day for 1 week significantly upregulated GPLD1 mRNA and protein expression in mice liver, however, one day of wheel running did not [26]. The author demonstrated that exercise upregulates 3-hydroxy butyrate in the liver, which promotes liver GPLD 1 expression [26].

Several factors could influence serum GPLD1 levels such as age, diabetes, liver dysfunction, statin therapy, and genetic variation in the GPLD1 gene [27–29]. We observed a significant correlation between plasma GPLD1 in different cohorts. Importantly, moderate exercise training in young adults did not change plasma GPLD1 levels (Fig 3), suggesting that age is one of the factors affecting GPLD1 levels. In addition, we found a positive correlation between age and plasma GPLD1 in elderly with normal glucose tolerance (Table 5). A previous study reported a negative correlation between GPLD1 and age in healthy and diseased subjects with a wide age range (24–89 yrs) [27]. A similar correlation between GPLD1 and age was also observed in non-alcoholic steatohepatitis (NASH) and healthy subjects (28–54 yrs) [14]. The authors showed that serum GPLD1 is significantly elevated in NASH patients and is not associated with body weight, BMI, waist-to-hip ratio, percent body fat, percent visceral fat, or visceral fat [14]. We found that BMI was positively correlated with GPLD1 in elderly subjects of the Oulu-45 cohort but not in young adults and prediabetic subjects. The positive correlation between GPLD1 and BMI was seen in elderly only with impaired glucose tolerance not in normal glucose tolerance and diabetes (Table 5). This could be due to the age of our young subjects (25 yrs) and the diseases status in prediabetic subjects, which was further confirmed in elderly with and without diabetes. Patients with non-alcoholic fatty liver diseases (NAFLD) had elevated serum GPLD1 levels and mRNA expression in the liver [14]. The authors demonstrated that GPLD1 increases diacylglycerol-acyltransferase homolog 1 (DGAT1), stearoyl coenzyme-A desaturase (SCD), and ATP citrate lyase (ACLY) expression in HepG2 cells enhancing de novo lipogenesis and serum triglyceride (TG) [14]. In addition, liver Gpld1 overexpressing mice showed a significant increase in fasting and postprandial TG and a reduction in TG-rich lipoprotein catabolism [30]. Obese rats displayed significantly higher serum GPLD1 concentration than lean rats [31]. An increase in plasma GPLD1 levels has been demonstrated in both animals and humans after the onset of diabetes and insulin resistance [17,18]. Sedentary diabetic rats significantly increased serum GPLD1 levels compared to the corresponding sedentary nondiabetic rats [32]. Several studies reported increased serum GPLD1 levels in STZ-induced diabetic rats [18], nonobese diabetic mice [12], and high-fructose diet-induced diabetic mice [30]. Increased liver GPLD1 expression has been shown in diabetic mice [12] and isolated β-cells of *ob*/*ob* mice [33]. We found an increased level of plasma GPLD1 in prediabetic subjects compared to healthy young adults and elderly subjects (Table 2). Our elderly subjects with diabetes showed significant increase in plasma GPLD1 (Table 3), suggesting that diabetes increases plasma GPLD1. In terms of the quantity and quality of PA, endurance exercise with moderate intensity has been suggested to induce beneficial effects, at least in part, by improving insulin profiles in diabetic rats [34]. Abdolmaleki & Heidarianpour found in streptozotocin-nicotinamide-induced diabetic rats that endurance training 5 days/week for 14 weeks decreased serum GPLD1 levels, which was negatively associated with the serum insulin level [32]. Serum GPLD1 levels positively correlated with prediabetes and were suggested as a novel biomarker for prediabetes in humans [35]. Serum GPLD1 levels were increased in type-1 diabetic patients and STZ-induced diabetic rats, and insulin administration reversed GPLD1 levels [17]. These studies indicate that exogenous insulin administration decreases GPLD1 synthesis in diabetic states leading to reduced serum GPLD1 levels. Resistance training (range 50%−100% of animal’s body weight with one-repetition maximum/3 days per week) in diabetic rats for 12 weeks decreased GPLD1 plasma levels [36]. Our results are consistent with these findings in our prediabetic subjects with reduced plasma GPLD1 in subjects with > 6000 daily steps compared to < 3000 steps/day. In addition, we found an inverse correlation between plasma GPLD1 levels and physical activity in elderly subjects.

The strength of our study is that we included a wide range of subjects from three different local cohorts consisting of young adults (25 yrs), prediabetic subjects (59.4 yrs), and elderly (68.9 yrs) with different PA intensities and durations. The study population consisted of individuals with similar ethnic and cultural backgrounds and comparable lifestyles from the same region in Northern Finland.

## Conclusion

Our study demonstrates that plasma GPLD1 levels vary with age, disease status and physical activity level. Diseases such as prediabetes and diabetes increase plasma GPLD1 levels. Average daily steps negatively correlated with plasma GPLD1 in elderly, however no change in prediabetic subjects performing physical activity for 3 months. Habitual physical activity did not alter plasma GPLD1 levels in individuals with prediabetes or diabetes. Age and level of physical activity might be the determining factor for plasma GPLD1 as young adults with moderate-intensity exercise did not change plasma GPLD1. Plasma GPLD1 did not change with blood pressure in all three study populations young adults, prediabetes and elderly subjects. Further human trials are required to study the effect of exercise on‌‌ plasma GPLD1 in different age subjects and diabetic people and its effect on cognition.

## Supporting information

S1 File S1-S3Table. Anthropological data and biochemical measurements of study population; young adults, elderly and prediabetes subjects.
(DOCX)

## References

[1] Chow LS, Gerszten RE, Taylor JM, Pedersen BK, van Praag H, Trappe S, et al. Exerkines in health, resilience and disease. Nat Rev Endocrinol. 2022;18(5):273–89. doi: 10.1038/s41574-022-00641-2 35304603 PMC9554896

[2] Livingston G, Huntley J, Sommerlad A, Ames D, Ballard C, Banerjee S, et al. Dementia prevention, intervention, and care: 2020 report of the Lancet Commission. Lancet. 2020;396(10248):413–46. doi: 10.1016/S0140-6736(20)30367-6 32738937 PMC7392084

[3] Erickson KI, Hillman C, Stillman CM, Ballard RM, Bloodgood B, Conroy DE, et al. Physical Activity, Cognition, and Brain Outcomes: A Review of the 2018 Physical Activity Guidelines. Med Sci Sports Exerc. 2019;51(6):1242–51. doi: 10.1249/MSS.0000000000001936 31095081 PMC6527141

[4] Sofi F, Valecchi D, Bacci D, Abbate R, Gensini GF, Casini A, et al. Physical activity and risk of cognitive decline: a meta-analysis of prospective studies. J Intern Med. 2011;269(1):107–17. doi: 10.1111/j.1365-2796.2010.02281.x 20831630

[5] Iso-Markku P, Aaltonen S, Kujala UM, Halme H-L, Phipps D, Knittle K, et al. Physical Activity and Cognitive Decline Among Older Adults: A Systematic Review and Meta-Analysis. JAMA Netw Open. 2024;7(2):e2354285. doi: 10.1001/jamanetworkopen.2023.54285 38300618 PMC10835510

[6] Guure CB, Ibrahim NA, Adam MB, Said SM. Impact of Physical Activity on Cognitive Decline, Dementia, and Its Subtypes: Meta-Analysis of Prospective Studies. Biomed Res Int. 2017;2017:9016924. doi: 10.1155/2017/9016924 28271072 PMC5320071

[7] Nagamatsu LS, Handy TC, Hsu CL, Voss M, Liu-Ambrose T. Resistance training promotes cognitive and functional brain plasticity in seniors with probable mild cognitive impairment. Arch Intern Med. 2012;172(8):666–8. doi: 10.1001/archinternmed.2012.379 22529236 PMC3514552

[8] Baker LD, Frank LL, Foster-Schubert K, Green PS, Wilkinson CW, McTiernan A, et al. Aerobic exercise improves cognition for older adults with glucose intolerance, a risk factor for Alzheimer’s disease. J Alzheimers Dis. 2010;22(2):569–79. doi: 10.3233/JAD-2010-100768 20847403 PMC3049111

[9] Norton S, Matthews FE, Barnes DE, Yaffe K, Brayne C. Potential for primary prevention of Alzheimer’s disease: an analysis of population-based data. Lancet Neurol. 2014;13(8):788–94. doi: 10.1016/S1474-4422(14)70136-X 25030513

[10] Northey JM, Cherbuin N, Pumpa KL, Smee DJ, Rattray B. Exercise interventions for cognitive function in adults older than 50: a systematic review with meta-analysis. Br J Sports Med. 2018;52(3):154–60. doi: 10.1136/bjsports-2016-096587 28438770

[11] Horowitz AM, Fan X, Bieri G, Smith LK, Sanchez-Diaz CI, Schroer AB, et al. Blood factors transfer beneficial effects of exercise on neurogenesis and cognition to the aged brain. Science. 2020;369(6500):167–73. doi: 10.1126/science.aaw2622 32646997 PMC7879650

[12] Deeg MA, Bowen RF, Williams MD, Olson LK, Kirk EA, LeBoeuf RC. Increased expression of GPI-specific phospholipase D in mouse models of type 1 diabetes. Am J Physiol Endocrinol Metab. 2001;281(1):E147-54. doi: 10.1152/ajpendo.2001.281.1.E147 11404232

[13] Qin W, Liang Y-Z, Qin B-Y, Zhang J-L, Xia N. The Clinical Significance of Glycoprotein Phospholipase D Levels in Distinguishing Early Stage Latent Autoimmune Diabetes in Adults and Type 2 Diabetes. PLoS One. 2016;11(6):e0156959. doi: 10.1371/journal.pone.0156959 27351175 PMC4925120

[14] Chalasani N, Vuppalanchi R, Raikwar NS, Deeg MA. Glycosylphosphatidylinositol-specific phospholipase d in nonalcoholic Fatty liver disease: a preliminary study. J Clin Endocrinol Metab. 2006;91(6):2279–85. doi: 10.1210/jc.2006-0075 16595594

[15] Jin J-K, Jang B, Jin HT, Choi E-K, Jung C-G, Akatsu H, et al. Phosphatidylinositol-glycan-phospholipase D is involved in neurodegeneration in prion disease. PLoS One. 2015;10(4):e0122120. doi: 10.1371/journal.pone.0122120 25867459 PMC4395093

[16] Kinoshita T. Biosynthesis and biology of mammalian GPI-anchored proteins. Open Biol. 2020;10(3):190290. doi: 10.1098/rsob.190290 32156170 PMC7125958

[17] Schofield JN, Stephens JW, Hurel SJ, Bell KM, deSouza JB, Rademacher TW. Insulin reduces serum glycosylphosphatidylinositol phospholipase D levels in human type I diabetic patients and streptozotocin diabetic rats. Mol Genet Metab. 2002;75(2):154–61. doi: 10.1006/mgme.2001.3287 11855934

[18] Kurtz TA, Fineberg NS, Considine RV, Deeg MA. Insulin resistance is associated with increased serum levels of glycosylphosphatidylinositol-specific phospholipase D. Metabolism. 2004;53(2):138–9. doi: 10.1016/j.metabol.2003.09.004 14767861

[19] Frisardi V, Solfrizzi V, Seripa D, Capurso C, Santamato A, Sancarlo D, et al. Metabolic-cognitive syndrome: a cross-talk between metabolic syndrome and Alzheimer’s disease. Ageing Res Rev. 2010;9(4):399–417. doi: 10.1016/j.arr.2010.04.007 20444434

[20] Juuti A-K, Hiltunen L, Rajala U, Laakso M, Härkönen P, Hedberg P, et al. Association of abnormal glucose tolerance with self-reported sleep apnea among a 57-year-old urban population in Northern Finland. Diabetes Res Clin Pract. 2008;80(3):477–82. doi: 10.1016/j.diabres.2008.02.002 18353486

[21] Leinonen A-M, Ahola R, Kulmala J, Hakonen H, Vähä-Ypyä H, Herzig K-H, et al. Measuring Physical Activity in Free-Living Conditions-Comparison of Three Accelerometry-Based Methods. Front Physiol. 2017;7:681. doi: 10.3389/fphys.2016.00681 28119626 PMC5222829

[22] Herzig K-H, Ahola R, Leppäluoto J, Jokelainen J, Jämsä T, Keinänen-Kiukaanniemi S. Light physical activity determined by a motion sensor decreases insulin resistance, improves lipid homeostasis and reduces visceral fat in high-risk subjects: PreDiabEx study RCT. Int J Obes (Lond). 2014;38(8):1089–96. doi: 10.1038/ijo.2013.224 24285336 PMC4125749

[23] Mäkelä KA, Leppäluoto J, Jokelainen J, Jämsä T, Keinänen-Kiukaanniemi S, Herzig K-H. Effect of Physical Activity on Plasma PCSK9 in Subjects With High Risk for Type 2 Diabetes. Front Physiol. 2019;10:456. doi: 10.3389/fphys.2019.00456 31114503 PMC6502968

[24] Stenbäck V, Leppäluoto J, Leskelä N, Viitala L, Vihriälä E, Gagnon D, et al. Step detection and energy expenditure at different speeds by three accelerometers in a controlled environment. Sci Rep. 2021;11(1):20005. doi: 10.1038/s41598-021-97299-z 34625578 PMC8501125

[25] Stenbäck V, Lehtonen I, Leppäluoto J, Gagnon D, Järvelin M-R, Tulppo M, et al. Associations of step accelerations and cardiometabolic risk markers in early adulthood. Eur J Public Health. 2025;35(1):128–33. doi: 10.1093/eurpub/ckae199 39656794 PMC11832146

[26] Ren T, He J, Zhang T, Niu A, Yuan Y, Zuo Y, et al. Exercise activates interferon response of the liver via Gpld1 to enhance antiviral innate immunity. Sci Adv. 2024;10(22):eadk5011. doi: 10.1126/sciadv.adk5011 38809975 PMC11804790

[27] Raymond FD, Fortunato G, Moss DW, Castaldo G, Salvatore F, Impallomeni M. Inositol-specific phospholipase D activity in health and disease. Clin Sci (Lond). 1994;86(4):447–51. doi: 10.1042/cs0860447 8168340

[28] Deeg MA, Raikwar NS, Johnson C, Williams CD. Statin therapy reduces serum levels of glycosylphosphatidylinositol-specific phospholipase D. Transl Res. 2007;150(3):153–7. doi: 10.1016/j.trsl.2007.03.008 17761367

[29] Deeg MA, Xuei X, Eckert G, Considine RV, Li YG, Pratt JH. Genetic variation of GPLD1 associates with serum GPI-PLD levels: a preliminary study. Biochim Biophys Acta. 2012;1821(3):381–5. doi: 10.1016/j.bbalip.2011.12.009 22260953

[30] Raikwar NS, Cho WK, Bowen RF, Deeg MA. Glycosylphosphatidylinositol-specific phospholipase D influences triglyceride-rich lipoprotein metabolism. Am J Physiol Endocrinol Metab. 2006;290(3):E463-70. doi: 10.1152/ajpendo.00593.2004 16219662

[31] Müller GA, Tschöp MH, Müller TD. Upregulated phospholipase D activity toward glycosylphosphatidylinositol-anchored proteins in micelle-like serum complexes in metabolically deranged rats and humans. Am J Physiol Endocrinol Metab. 2020;318(4):E462–79. doi: 10.1152/ajpendo.00504.2019 31961708

[32] Abdolmaleki F, Heidarianpour A. Endurance exercise training restores diabetes-induced alteration in circulating Glycosylphosphatidylinositol-specific phospholipase D levels in rats. Diabetol Metab Syndr. 2020;12:43. doi: 10.1186/s13098-020-00553-z 32467736 PMC7236206

[33] Bowen RF, Raikwar NS, Olson LK, Deeg MA. Glucose and insulin regulate glycosylphosphatidylinositol-specific phospholipase D expression in islet beta cells. Metabolism. 2001;50(12):1489–92. doi: 10.1053/meta.2001.28087 11735099

[34] Nazem F, Farhangi N, Neshat-Gharamaleki M. Beneficial Effects of Endurance Exercise with Rosmarinus officinalis Labiatae Leaves Extract on Blood Antioxidant Enzyme Activities and Lipid Peroxidation in Streptozotocin-Induced Diabetic Rats. Can J Diabetes. 2015;39(3):229–34. doi: 10.1016/j.jcjd.2014.11.003 25659282

[35] von Toerne C, Huth C, de Las Heras Gala T, Kronenberg F, Herder C, Koenig W, et al. MASP1, THBS1, GPLD1 and ApoA-IV are novel biomarkers associated with prediabetes: the KORA F4 study. Diabetologia. 2016;59(9):1882–92. doi: 10.1007/s00125-016-4024-2 27344311

[36] Heidarianpour A, Keshvari M, Shahidi S, Zarei M. Modulation of GPC-4 and GPLD1 serum levels by improving glycemic indices in type 2 diabetes: Resistance training and hawthorn extract intervention. Heliyon. 2023;9(4):e15537. doi: 10.1016/j.heliyon.2023.e15537 37151681 PMC10161711

