# Supplementary material for: Physical activity and glycosylphosphatidylinositol-specific phospholipase D1 (GPLD1) plasma levels in different age cohorts
Source: PLoS One. 2026 Jun 1;21(6):e0349883. doi: 10.1371/journal.pone.0349883 (PMC13225414; doi:10.1371/journal.pone.0349883)
Supplement: S1 File — Anthropological data and biochemical measurements of study population; young adults, elderly and prediabetes subjects. (DOCX) [file pone.0349883.s001.docx]

**Physical activity and glycosylphosphatidylinositol-specific phospholipase D1 (GPLD1) plasma levels in different age Cohorts**

Ghulam Shere Raza^1^, Jari Jokelainen^2,3^, Ville Stenbäck^1^, Nalini Sodum^1^, Toni Karhu^1^, Dominique D. Gagnon^4,5^, Juhani Leppäluoto^1^, Marjo-Riitta Järvelin^2,6^, Sirkka Keinänen-Kiukaanniemi^2,7^, Karl-Heinz Herzig ^1,8^

**Supporting Information**

**S1 Table. Anthropological data of study population 1; young adults.** Values are presented as mean ± SD.

|  | **Cold** | **Thermoneutral** | **All** |
| --- | --- | --- | --- |
| **n** | **15** | **19** | **34** |
| Age | 24.7 ± 3.2 | 24.4 ± 3.5 | 24.6 ± 3.3 |
| Height (cm) | 177.0 ± 12.1 | 172.0 ± 10.3 | 174.2 ± 11.2 |
| Body mass (kg) | 80.3 ± 17.9 | 77.4 ± 12.2 | 78.7 ± 14.8 |
| BMI (kg/cm^2^) | 25.4 ± 3.8 | 26.2 ± 3.4 | 25.8 ± 3.5 |
| BF % | 27.3 ± 10.0 | 30.3 ± 10.7 | 28.9 ± 10.3 |
| FM (kg) | 22.4 ± 11.9 | 23.5 ± 9.3 | 23.0 ± 10.4 |
| FFM (kg) | 57.9 ± 12.9 | 53.9 ± 11.2 | 55.7 ± 12.0 |
| VO2 peak (ml/kg/min) | 42.8 ± 9.4 | 40.5 ± 9.5 | 41.3 ± 9.4 |
| W_max_ | 265 ± 60 | 246 ± 60 | 254 ± 60 |

BMI; body mass index, BF %; body fat percent, FM; fat mass, FFM; fat free mass, VO2 peak; peak oxygen uptake, W_max_; maximal cycling power in the VO2 peak test.

**S2 Table. Anthropological and biochemical measurements of study population 2; elderly (Oulu 1945).** Means and standard deviations in brackets or numbers and percentage of subjects (*n*; %) are shown.

|  | **Total** | **NGT** | **IGT** | **T2DM** | ***p*-Value** |
| --- | --- | --- | --- | --- | --- |
| **n** | **526** | **275** | **172** | **79** |  |
| Age | 68.9 (0.55) | 69.0 (0.56) | 68.9 (0.51) | 68.9 (0.50) | 0.441 |
| Sex |  |  |  |  | **0.019** |
| Men | 225 (41.9%) | 108 (39.3%) | 72 (41.9%) | 45 (57.0%) |  |
| Women | 301 (58.1%) | 167 (60.7%) | 100 (58.1%) | 34 (43.0%) |  |
| BMI (kg/m^2^) | 27.7 (4.71) | 26.1 (3.63) | 28.4 (4.74) | 30.9 (5.25) | **<0.001** |
| Normal | 159 (30.3%) | 112 (40.8%) | 39 (22.6%) | 9 (11.5%) |  |
| Overweight | 232 (44.1%) | 130 (47.2%) | 74 (43.3%) | 28 (35.6%) |  |
| Obese | 135 (25.6%) | 33 (12.0%) | 59 (34.1%) | 42 (52.9%) |  |
| Waist circumference (cm) | 94.0 (13.7) | 88.9 (11.5) | 97.0 (12.8) | 105 (14.0) | **<0.001** |
| Fasting glucose (mmol/L) | 5.75 (1.03) | 5.31 (0.41) | 5.88 (0.55) | 7.02 (1.85) | **<0.001** |
| Fasting insulin (pmol/L) | 15.4 (17.0) | 11.3 (5.62) | 16.1 (9.42) | 27.8 (37.3) | **<0.001** |
| HOMA2-B | 109 (42.0) | 108 (33.7) | 112 (42.9) | 106 (62.1) | 0.406 |
| HOMA2-S | 71.7 (39.9) | 82.4 (38.7) | 63.2 (38.4) | 52.3 (35.8) | **<0.001** |
| HOMA2-IR | 1.91 (1.36) | 1.48 (0.71) | 2.12 (1.20) | 2.98 (2.42) | **<0.001** |
| DP (mmHg) | 85.6 (9.73) | 84.1 (9.50) | 88.1 (9.44) | 85.2 (10.1) | **<0.001** |
| SP (mmHg) | 144 (17.7) | 141 (17.2) | 148 (18.0) | 147 (16.6) | **<0.001** |
| Daily steps | 8869 (3696) | 9283 (3613) | 8499 (3686) | 8118 (3885) | **0.024** |
| Physical activity questionnaire |  |  |  |  | **<0.001** |
| Non-active ^a^ | 123 (23.3%) | 50 (18.1%) | 37 (21.4%) | 35 (44.6%) |  |
| Active ^b^ | 403 (76.7%) | 225 (81.9%) | 135 (78.6%) | 44 (55.4%) |  |
| Smoking: |  |  |  |  | **0.001** |
| Current | 65 (12.4%) | 31 (11.4%) | 16 (9.5%) | 17 (21.8%) |  |
| Former (>6 months earlier) | 181 (34.4%) | 86 (31.2%) | 61 (35.5%) | 34 (42.6%) |  |
| Never | 280 (53.2%) | 158 (57.4%) | 95 (55.0%) | 28 (35.6%) |  |
| Alcohol (g/d) | 1.85 (4.61) | 1.23 (2.29) | 2.80 (7.11) | 1.99 (3.37) | **<0.001** |
| Total cholesterol (mmol/L) | 5.33 (1.22) | 5.44 (1.15) | 5.51 (1.18) | 4.60 (1.26) | **<0.001** |
| HDL-C (mmol/L) | 1.65 (0.46) | 1.74 (0.46) | 1.61 (0.43) | 1.40 (0.38) | **<0.001** |
| Triglycerides (mmol/L) | 1.27 (0.81) | 1.07 (0.42) | 1.42 (0.70) | 1.63 (1.53) | **<0.001** |
| HbA1c (mmol/L) | 40.6 (5.87) | 38.9 (3.45) | 40.2 (4.00) | 47.1 (9.95) | **<0.001** |
| FLI | 45.6 (28.9) | 33.2 (23.9) | 54.5 (28.0) | 68.0 (25.9) | **<0.001** |
| h-CRP (mg/L) | 3.49 (9.47) | 2.30 (4.42) | 4.49 (12.7) | 5.36 (13.0) | **0.002** |
|  |  |  |  |  |  |

HOMA2-B, HOMA2-S and HOMA2-IR: homeostatic model assessment for beta-cell function, insulin sensitivity, and insulin resistance, respectively, DP; diastolic blood pressure; SP: systolic blood pressure ^a^; <30 min light exercise, e.g., walking/week, ^b^; >30 min light exercise, e.g., walking/at least once a week, HDL-C; high-density lipoprotein cholesterol, HbA1c; hemoglobin A1c, FLI; fatty liver index, h-CRP; high-sensitivity C-reactive protein. *p*-values < 0.05 are considered significant (in bold format).

**S3 Table. Anthropological and biochemical measurements of study population 3; Prediabetes subjects before and after a 3-month physical activity intervention.** Means and standard deviations in brackets are shown.

|  | ***Intervention*** | | **P** | ***Control*** | | **P** | ***Difference* ^a^** | ***ANOVA*^b^ (p*-value)*** | | |
| --- | --- | --- | --- | --- | --- | --- | --- | --- | --- | --- |
| **n** | ***33*** | |  | ***35*** | |  |  |  | | |
|  | ***Baseline*** | ***After 3 months*** |  | ***Baseline*** | ***After 3 months*** |  |  | *Model 1* | *Model 2* | *Model 3* |
| Fasting glucose (mmol l^−1^) | 6.6 (0.8) | 6.4 (0.8) | **0.019** | 6.5 (0.7) | 6.6 (1.8) | 0.659 | 0.3 (−0.2 to 0.9) | 0.256 | 0.2183 | 0.247 |
| 2-h glucose (mmol l^−1^) | 8.9 (2.6) | 7.8 (2.6) | **0.017** | 8.7 (2.6) | 7.9 (3.0) | 0.061 | 0.2 (−0.9 to 1.3) | 0.915 | 0.831 | 0.789 |
| Fasting insulin (mU l^−1^) | 18.7 (11.0) | 15.6 (10.3) | **0.008** | 16.3 (11.9) | 16.9 (15.9) | 0.757 | 3.4 (−0.8 to 7.6) | **0.047** | **0.041** | **0.035** |
| 2-h insulin (mU l^−1^) | 116.9 (70.8) | 75.6 (62.7) | **<0.001** | 94.8 (72.8) | 91.4 (60.6) | 0.794 | 26.6 (1.1 to 51.8) | **0.003** | **0.003** | **0.003** |
| Homa-IR | 5.7 (3.8) | 4.6 (3.3) | **0.003** | 4.8 (3.7) | 4.9 (4.4) | 0.939 | 1.0 (−0.2 to 2.2) | **0.050** | **0.043** | **0.036** |
| Total cholesterol (mmol l^−1^) | 5.3 (0.8) | 5.0 (0.8) | **0.001** | 5.7 (1.3) | 5.5 (1.1) | 0.298 | 0.3 (−0.0 to 0.7) | **0.064** | **0.062** | **0.041** |
| HDL-C (mmol l^−1^) | 1.4 (0.3) | 1.4 (0.4) | 0.514 | 1.5 (0.3) | 1.5 (0.3) | 0.544 | 0.0 (−0.1 to 0.1) | 0.674 | 0.660 | 0.553 |
| LDL-C (mmol l^−1^) | 3.0 (0.8) | 3.0 (0.8) | 0.771 | 3.2 (1.2) | 3.6 (1.0) | **0.018** | 0.4 (0.1 to 0.7) | **0.014** | **0.012** | **0.008** |
| Triglycerides (mmol l^−1^) | 1.7 (0.6) | 1.5 (0.4) | **<0.001** | 1.8 (0.7) | 1.7 (0.9) | 0.391 | 0.2 (−0.0 to 0.5) | 0.239 | 0.251 | 0.179 |
| Systolic BP (mm Hg) | 138.5 (16.4) | 131.3 (15.2) | **0.005** | 150.4 (20.2) | 139.0 (16.0) | **<0.001** | 1.0 (−5.4 to 7.5) | 0.688 | 0.653 | 0.742 |
| Diastolic BP (mm Hg) | 83.8 (8.0) | 79.1 (8.2) | **0.004** | 85.4 (9.5) | 80.1 (8.8) | **0.007** | 0.4 (−3.5 to 4.4) | 0.820 | 0.479 | 0.392 |
| Max. O_2_ uptake (ml kg^−1^ min^−1^) | 22.7 (4.6) | 26.3 (6.8) | **0.002** | 23.6 (4.8) | 26.4 (5.5) | **0.002** | −0.9 (−3.0 to 1.2) | 0.394 | 0.450 | 0.428 |
| Weight (kg) | 92.4 (19.4) | 91.5 (20.3) | **0.028** | 84.6 (14.4) | 83.3 (15.3) | **0.047** | −0.1 (−1.6 to 1.4) | 0.899 | 0.970 |  |
| BMI (kg m^−2^) | 32.6 (5.7) | 32.3 (6.1) | **0.038** | 30.9 (4.8) | 30.2 (4.9) | **0.011** | −0.3 (−0.8 to 0.3) | 0.379 | 0.402 |  |
| Waist circumference (cm) | 97.4 (17.2) | 95.1 (14.1) | **0.061** | 91.4 (9.7) | 88.3 (8.4) | **0.005** | −2.3 (−4.9 to 0.4) | 0.100 | 0.123 | 0.132 |
| Body fat (%) | 40.0 (7.6) | 37.9 (9.0) | **0.018** | 38.6 (9.0) | 37.5 (9.3) | **0.020** | 0.9 (−1.0 to 2.8) | 0.279 | 0.284 | 0.199 |
| Visceral fat area (cm^2^) | 163.7 (29.8) | 155.9 (31.4) | **0.013** | 156.3 (28.0) | 154.0 (32.7) | 0.185 | 5.5 (−1.4 to 12.3) | 0.073 | 0.082 | **0.030** |
| Skeletal mass (kg) | 29.6 (8.4) | 30.4 (9.3) | 0.140 | 28.5 (5.5) | 28.6 (5.3) | 0.867 | −0.7 (−1.9 to 0.4) | 0.443 | 0.328 | 0.214 |

ANOVA; analysis of variance, Homa-IR; homeostasis model assessment-estimated insulin resistance, HDL-C; high-density lipoprotein cholesterol, LDL-C; low-density lipoprotein cholesterol, Max; maximum, T2D; type 2 diabetes. Data is mean and standard deviation (s.d.) in brackets or 95% confidence interval. *P* is paired *t*-test probability. Model 1=controlled with baseline value. Model 2=Model 1+sex+age. Model 3=Model 2+weight change during intervention.
